# Colostomy as a definitive treatment in an ALS patient with acute colonic Pseudo-obstruction refractory to medical management, a case report

**DOI:** 10.1186/s12883-022-02893-x

**Published:** 2022-09-22

**Authors:** Guanghao Liu, Jennifer Hrabe, Rolando Sanchez

**Affiliations:** 1 Department of Neurology, University of Iowa Hospitals and Clinics, Iowa City, 52333 USA; 2 Department of Surgery, Division of Gastrointestinal, Minimally invasive and Bariatric Surgery, University of Iowa Hospitals and Clinics, Iowa City, 52333 USA; 3 Department of Internal Medicine, Division of Pulmonary, Critical Care and Occupational Medicine, University of Iowa Hospitals and Clinics, Iowa City, 52333 USA

**Keywords:** ALS, Colostomy, Acute colonic pseudo-obstruction

## Abstract

**Background:**

Amyotrophic lateral sclerosis (ALS) is a fatal motor neuron disease, and ALS patients may experience disturbed gastrointestinal motility often resulting in acute colonic pseudo-obstruction (ACPO). There is currently a paucity in the literature to guide the treatment of patients with both ALS and ACPO.

**Case presentation:**

Here we describe a 39-year-old male patient with advanced ALS who developed ACPO. His condition was refractory to both medical and procedural managements including polyethylene glycol, senna, and docusate suppository, metoclopramide, linaclotide, erythromycin, prucalopride, neostigmine, and repeated colonoscopies. He ultimately underwent successful colostomy for palliation. Here we report the peri-operative multidisciplinary approach taken with this case, the surgical procedures, the potential risks, and the outcome.

**Conclusion:**

The patient is delighted with the result and requested publication of this case to raise awareness of constipation in ALS patients and promote the consideration of colostomy as a treatment option for patients with ileus resistant to conservative management. Ultimately, a multidisciplinary team approach is required to properly assess the risks and benefits to achieve good clinical outcomes.

## Background

Amyotrophic lateral sclerosis (ALS) is a motor neuron disease that is fatal and characterized by degenerative changes in both upper and lower motor neurons [1]. The disease usually starts in late middle life and progresses relentlessly with muscle atrophy and weakness. Death usually occurs within 3 years of onset from respiratory failure [2]. ALS is the most common adult motor neuron disease with an incidence of 2 per 100,000 and prevalence of 5.4 per 100,000 individuals [3]. Besides motor nerve dysfunction, constipation is also a common complication in ALS patients, especially in later stages of the disease. One pilot study reported an increase from 33 to 65% in constipation symptoms after diagnosis of ALS in a cohort of 66 patients [4].

Besides motor dysfunction, ALS patients may experience disturbed gastrointestinal motility due to dehydration, decreased nutrition or low dietary fiber intake, age-related deconditioning/inactivity, autonomic dysregulation of the digestive and orthostatic homeostasis, and subconscious hesitation to move bowels related to deconditioning. The impaired motility is usually due to intestinal pseudo-obstruction, including acute colonic pseudo-obstruction (ACPO), rather than by mechanical obstruction. ACPO, also known as Ogilvie’s syndrome, is characterized by a sudden onset of massive colonic dilation without mechanical obstruction. There is little published to guide the treatment of patients with both ALS and ACPS. Here we describe a patient with advanced ALS who developed ACPO refractory to medical management and who ultimately underwent colostomy for palliation. Treatment was successful, and the patient wishes to raise awareness of this treatment option for the public. It is important to balance the risks of this treatment against its benefit and emphasize the multidisciplinary team approach required.

## Case presentation

**Fig. 1:**
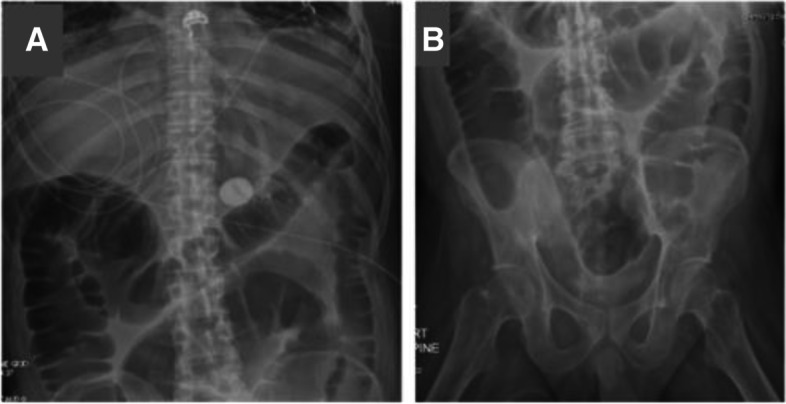
Abdominal X-ray documenting the course of the ileus. **A**, **B** Abdominal x-rays showing dilated large intestine bowel loops without any discernable obstructions

Our patient was a 39-year-old male with a past medical history of ALS (diagnosed at age 30) with quadriplegia, tracheostomy tube reliant on mechanical ventilation, and percutaneous endoscopic gastrostomy (PEG) tube for enteral nutrition (with no history of bowel dysmotility at baseline). He originally presented to an outside hospital for bacterial pneumonia. He subsequently went into pulseless electrical activity and ventricular fibrillation arrest. Return of spontaneous circulation was achieved with pulmonary cardiac resuscitation and the patient was maintained on vasopressors for the next several days. During this time, he did not receive any enteral nutrition and did not pass bowel movements. He was transferred to our facility for higher level of care and long-term tracheostomy. Upon arrival to our institution, his abdomen was severely tense and distended. Neurologically he was awake and alert but could only raise his eyebrows to answer questions and communicates by using a visual trackpad device. He was completely bed bound and paraplegic with significant muscle atrophy and fasciculations. Sensations were intact throughout. Admission abdominal X-ray demonstrated air-filled, dilated large intestine (Fig. 1A, B). Abdominal compartment syndrome was ruled out due to normal bladder pressure and an acute colonic obstruction was ruled out with colonoscopy, thus leading to the diagnosis of ACPO, which is characterized by a sudden onset of massive colonic dilation without mechanical obstruction.

Medications used to treat the initial colonic ileus included polyethylene glycol 17 g twice a day, sennosides 8.5 mg twice a day, metoclopramide and bisacodyl suppository 10 mg as needed and fleet enemas as needed, all with minimal stool output for the initial 2 weeks of hospital admission. In isolated case reports, neostigmine has been shown to have some efficacy in treating ALS patients with non-obstructive ileus [5]. Neostigmine (2 mg subcutaneous injection over 30 minutes) was administered to this patient three times with temporary improvement in stool output and decrease in abdominal distention. However, each time it was administered, the ileus would return a few days later. The patient also underwent several decompressive colonoscopies and had a rubber rectal tube placed as a temporary decompression measure. We then switched from metoclopramide to erythromycin 400 mg daily. We also wanted to add lubiprostone 24 μg BID as well, but it could not be crushed and given by PEG tube, so lubiprostone was switched to linaclotide 145 mg daily. The combination medications provided some transient success in allowing the patient to have small bowel movements and partially relieved abdominal distention. Unfortunately, the patient developed distended abdomen again with nausea and vomiting after 4 days on this regimen. At that time, the culprit of nausea/vomiting was attributed to linaclotide, so it was switched to prucalopride 2 mg daily. On this new regimen, patient had a few spontaneous albeit smaller bowel movements but still required frequent manually or colonoscopy decompressions to relieve abdominal distensions. The entire medication trial period lasted for nearly 4 weeks and was associated with significant patient frustration and discomfort. He also had limited enteral feeding tolerance from his PEG tube, which required frequent pausing of feeds throughout this period. Right before the surgery, he received several days of total parenteral nutrition as well. Due to the recurrent and refractory nature of the patient’s ileus, while acknowledging the risk of anesthesia in the setting of ALS, the surgical team offered to perform a colostomy. Finally, a sigmoid colon colostomy was performed under general anesthesia using the tracheostomy and via a minimally invasive, laparoscopic approach.

The patient was brought to the operating room and placed supine. Lines and monitors were placed. A preinduction huddle was conducted. He has a tracheostomy in place and general anesthesia was induced. He was positioned split leg with arms tucked and pressure points padded. A surgical timeout was held. A digital exam was first performed after removal of the rectal tube. A flexible sigmoidoscope was then inserted into the rectum and sigmoid in attempt to suction out any remaining bowel prep and gas. This was done to facilitate laparoscopy. The abdomen was then prepped and draped in a sterile fashion. A vertical incision above the umbilicus was made with a knife and deepened with electrocautery. The fascia was grasped and elevated and incised and a 12 mm trocar was placed. Pneumoperitoneum was induced, which he tolerated. The camera was placed and no injury with trocar placement was identified. Under direct vision, 5 mm trocars were placed in the right mid and lower quadrants. That right lower quadrant trocar was later upsized to a 12 mm trocar to facilitate laparoscopic stapling. He was positioned Trendelenburg with right side down.

The sigmoid colon was grasped and traced out cephalad and caudad. It appeared that we had enough redundancy in the sigmoid colon to use this as a colostomy. A point of sigmoid was chosen to use for the colostomy. A window was made in the mesentery with judicious electrocautery and blunt dissection. The bowel was divided with an endo GIA purple load staples using 2 firings. The mesentery was divided with serial firings of the LigaSure. The distal stump upon inspection appeared to be ischemic for several centimeters, so we re-divided that distal segment at a point of viability. Ultimately, we collected the portion of the colon that was excised, and this was passed off the field. The mesentery was divided to facilitate reach. This was done with LigaSure. Once we had adequate mobilization, a circular disc of skin was excised with electrocautery at a pre-marked spot. The subcutaneous and anterior fascia was incised vertically, the rectus was split, the posterior sheath was incised. The short segment of resected sigmoid colon was passed through and passed off the field, and the end of the colon was passed to be matured through that orifice. The abdomen was then re-insufflated, and a transversus abdominis plane block was performed on each side of the abdomen. The right lower quadrant 12 mm trocar site fascia was reapproximated with a GraNee needle using a 0 Vicryl suture in a figure-of-eight configuration. The abdomen was de-sufflated and the umbilical trocar site was closed with a 0 Vicryl in a figure-of-eight. All skin wounds were sewn with 4–0 Monocryl in a running fashion and skin glue was applied. The colostomy was then matured in the usual fashion. The patient was taken to ICU in stable condition. There were no complications.

After colostomy construction, the patient had brown ostomy output by post-operative day (POD) 3, indicating return of bowel function. Enteral feeds by PEG tube were started by POD 2 and reached goal by POD 5. The patient’s postoperative course was uncomplicated, and he was discharged home by POD 7. He was continued on the prucalopride 2 mg daily and erythromycin 400 mg three times daily regimen since this combination had provided him the most benefit prior to the surgery. One and a half year after the surgery, the patient continues to do well on this regimen and is currently achieving his goal of staying out of the hospital and spending time at home with family.

## Discussion and conclusions

ALS is characterized as a fatal neurodegenerative disease of both upper and lower motor neurons at various spinal levels, with symptoms such as limb weakness and progressive speech and respiratory failure. However, mounting evidence suggests manifestations of ALS extend beyond the anterior horn cells and corticospinal tracts. Symptoms associated with autonomic dysregulation such as urinary or gastrointestinal dysfunction, and orthostatic intolerance have been reported in over 25% of ALS patients [6–8]. While causes of impaired bowel function include common etiology such as dehydration, inadequate dietary fiber intake, and inactivity, other etiologies may contribute. For example, hyperfunction of the adrenergic sympathetic nervous system may also contribute to splanchnic hypoperfusion [9], which in-turn, may lead to worsening intestinal hypoperistalsis, increased transition time, and slowed gastrointestinal motility.

Acute colonic pseudo-obstruction (ACPO), also known as Ogilvie’s syndrome, often manifest as a rare complication after surgery with cecal and colon dilatation without mechanical or anatomical obstruction. Its etiology is not well understood and is hypothesized to be secondary to parasympathetic dysfunction [10] resulting in erratic peristalsis and progressive dilatation [11]. This condition occurs more commonly in patients after hip, cardiac surgery, renal transplantation, or those after retroperitoneal/bowel operations. Our ALS patient developed colonic ileus after a pulseless electrical activity and ventricular fibrillation arrest, which did not fit into the more common causes of ACPO. It is possible that the hypoxia resulting from cardiac arrest may have damaged the autonomic nerve fibers responsible for promoting colonic motility. Our patient also has motor neuron disease which caused pelvic floor muscles dysfunction as shown by the dilation of the entire colon seen in Fig. 1 A, B. Taken together, these reasons may be why our patient did not respond to the commonly used treatments in post op ACPO patients with normal neuromuscular axis. There is currently a paucity in the literature to guide treatment in rare cases of ALS patients with ACPO who do not respond to traditional treatments of laxatives plus various pro-motility agents and repeated endoscopic decompressions. Although neostigmine can be used to increase intestinal motility, its side effect of bradycardia and hypotension can be problematic and necessitates close monitoring when administering the drug [5].

Another important consideration in determining whether to proceed with surgery in our patient was weighing the anesthetic risks since ALS patients have increased complications from weakened respiratory muscles with the risks highest for patients without tracheostomies. In these patients, a safer approach from a respiratory standpoint would be either using spinal anesthetic and open colostomy versus a diverting colostomy under local anesthesia. Since our patient already had tracheostomy, his colostomy was performed under general anesthesia using a minimally invasive, laparoscopic approach. Our patient finally had resolution of the ileus after colostomy and returned to normal enteral feeds and bowel function by POD 5. There is another case report describing an ALS patient who elected to receive a prophylactic “triple-ostomy,” which included an end colostomy, a suprapubic catheter, and a percutaneous gastrostomy with good results and significant improvement in the patient’s quality of life [12]. However, it is important to note that the rate of surgical management in ACPO is very, very low overall with colostomy often considered as the last resort in cases refractory to conservative pharmacologic management due to its higher morbidity and mortality rates [13].

Ultimately, our patient wanted his case to be published to raise awareness of constipation in ALS patients and promote the consideration of colostomy as a treatment option for patients with ileus resistant to conservative management. However, it is important to balance the potential benefits and risks of the surgical procedure and emphasize the multidisciplinary team approach that is required to achieve good clinical outcomes.

## Data Availability

Not applicable since no data was generated in this study.

## Abbreviations

ALS: Amyotrophic lateral sclerosis
ACPO: Acute colonic pseudo-obstruction
POD: Post operative day
PEG: Percutaneous endoscopic gastrostomy
